# Singlet Fission‐Based High‐Resolution X‐Ray Imaging Scintillation Screens

**DOI:** 10.1002/advs.202300406

**Published:** 2023-04-21

**Authors:** Jian‐Xin Wang, Jun Yin, Luis Gutiérrez‐Arzaluz, Simil Thomas, Wenyi Shao, Husam N. Alshareef, Mohamed Eddaoudi, Osman M. Bakr, Omar F. Mohammed

**Affiliations:** ^1^ Advanced Membranes and Porous Materials Center Division of Physical Science and Engineering King Abdullah University of Science and Technology Thuwal 23955‐6900 Kingdom of Saudi Arabia; ^2^ KAUST Catalysis Center Division of Physical Sciences and Engineering King Abdullah University of Science and Technology Thuwal 23955‐6900 Kingdom of Saudi Arabia; ^3^ Department of Applied Physics The Hong Kong Polytechnic University Hong Kong 999077 P. R. China; ^4^ Materials Science and Engineering Physical Science and Engineering Division King Abdullah University of Science and Technology (KAUST) Thuwal 23955‐6900 Saudi Arabia

**Keywords:** energy transfer, high‐resolution X‐ray imaging scintillator, singlet fission

## Abstract

X‐ray imaging technology is critical to numerous different walks of daily life, ranging from medical radiography and security screening all the way to high‐energy physics. Although several organic chromophores are fabricated and tested as X‐ray imaging scintillators, they generally show poor scintillation performance due to their weak X‐ray absorption cross‐section and inefficient exciton utilization efficiency. Here, a singlet fission‐based high‐performance organic X‐ray imaging scintillator with near unity exciton utilization efficiency is presented. Interestingly, it is found that the X‐ray sensitivity and imaging resolution of the singlet fission‐based scintillator are dramatically improved by an efficient energy transfer from a thermally activated delayed fluorescence (TADF) sensitizer, in which both singlet and triplet excitons can be efficiently harnessed. The fabricated singlet fission‐based scintillator exhibits a high X‐ray imaging resolution of 27.5 line pairs per millimeter (lp mm^−1^), which exceeds that of most commercial scintillators, demonstrating its high potential use in medical radiography and security inspection.

## Graphical abstract

A novel singlet fission‐based organic X‐ray imaging scintillator is presented, whose X‐ray sensitivity and imaging resolution are greatly improved by efficient energy transfer from a thermally activated delayed fluorescence (TADF) sensitizer. More importantly, the high X‐ray imaging resolution of 27.5 lp mm^−1^ exceeds most reported organic and commercial scintillators.

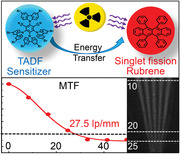

## 1 Introduction

X‐ray‐responsive materials, commonly known as scintillators, can convert ionizing irradiation into visible photons, and they have been widely used for real‐world technological applications, including in medical radiography, the food industry, and security inspection.^[^ ^1^, ^2^, ^3^, ^4^, ^5^, ^6^, ^7^, ^8^, ^9^ ^]^ Currently, researchers in various disciplines are dedicating tremendous efforts to exploring and optimizing the composition and synthesis of perovskite and ceramic materials to obtain high‐performance scintillators, but the inherent shortcomings of their harsh preparation conditions, poor air and light stability, and high fabrication cost limits their sustainable development.^[^ ^10^, ^11^, ^12^, ^13^, ^14^, ^15^, ^16^, ^17^, ^18^, ^19^, ^20^, ^21^ ^]^ Organic scintillators, in contrast, exhibit low toxicity, high stability, good mechanical flexibility, and abundant radioluminescence (RL) mechanisms.^[^ ^22^, ^23^, ^24^, ^25^, ^26^, ^27^, ^28^, ^29^, ^30^ ^]^ However, the low X‐ray absorption cross‐section and inefficient exciton utilization efficiency of most organic scintillators lead to poor X‐ray sensitivity and low spatial‐imaging resolution. Therefore, the search for new photoactive organic materials with high X‐ray to photon conversion efficiency for high‐performance X‐ray imaging scintillators is urgently needed.

**Figure 1:**
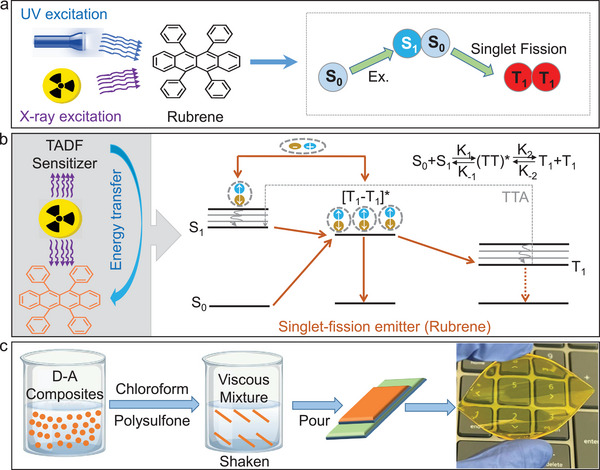
a) Singlet fission process of rubrene. b) Mechanism by which the radioluminescence (RL) efficiency of the singlet fission‐based scintillator is significantly improved by the efficient energy transfer from the TADF‐Br sensitizer. c) Illustration of the preparation procedure of the transparent scintillation screens. Ex, excitation; S, singlet; T, triplet; TADF, thermally activated delayed fluorescence; TTA, triplet–triplet annihilation.

Singlet fission is one of the viable approaches to exceed the maximum solar‐to‐electric power conversion efficiency (Shockley–Queisser limit) of conventional solar cells.^[^ ^31^, ^32^, ^33^, ^34^, ^35^, ^36^, ^37^, ^38^, ^39^ ^]^ Singlet fission is a spin‐allowed photophysical process in which a photoexcited singlet (S_1_) state generates two triplet states (T_1_), yielding an individual triplet yield (*φ*_T_) of over 100% (**Figure** **1**a).^[^ ^40^ ^]^ Moreover, singlet fission involves a multiexciton state (triplet pair ^1^(TT)) with an overall singlet‐spin multiplicity.^[^ ^41^, ^42^, ^43^ ^]^ The “super‐exchange”^[^ ^44^ ^]^ between the S_1_ and ^1^(TT) pair allows the luminescence of singlet fission material to encompass both excited states, making it possible to utilize both singlet and triplet excitons upon X‐ray excitation. In addition, the energy level of the S_1_ excited state of singlet fission materials is twice that of its triplet excited state; so, the process of triplet–triplet annihilation (TTA) may happen, which further improves the utilization of excitons under X‐ray excitation.^[^ ^44^ ^]^ In other words, the non‐radiative triplet state converses back to the luminescent singlet state through TTA, thereby further improving the luminous efficiency (Figure 1b). Therefore, singlet fission materials may be one of the best candidates for high‐performance organic X‐ray imaging scintillators, which are of great interest to researchers in various fields.

Here, we present a novel singlet fission‐based scintillator with high X‐ray sensitivity and spatial imaging resolution. Moreover, the scintillation performance of the singlet fission‐based scintillator was further improved by an efficient energy transfer^[^ ^25^, ^45^, ^46^, ^47^, ^48^ ^]^ from a thermally activated delayed fluorescence (TADF) sensitizer with eight Br atoms, in which both singlet and triplet excitons could be efficiently harnessed. More importantly, the singlet fission‐based scintillator exhibited a high X‐ray imaging resolution of 27.5 line pairs per millimeter (lp mm^−1^), exceeding most commercial scintillators. We believe that our findings will serve as a benchmark for the fabrication of efficient organic scintillators from materials beyond ceramics and perovskites.

## 2 Results and Discussion

**Figure 2:**
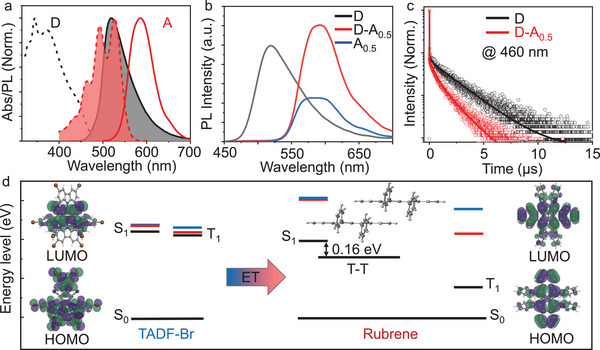
a) Normalized absorption (dash line) and emission (solid line) spectra of TADF‐Br (D) and rubrene (A) doped in polysulfone (PSF) at 5 wt% and 0.5 wt%, respectively. b) Emission spectra of D, D–A_0.5_, and A_0.5_ composite films, and c) the corresponding emission decay profiles of D and D–A_0_._5_ films collected at 460 nm. d) The electronic cloud distributions for lowest unoccupied molecular orbital (LUMO) and the highest occupied molecular orbital (HOMO), and energy levels of D and A, respectively (the blue and red line represent the higher singlet and triplet excited states, respectively).

Doping rubrene into polysulfone (PSF) polymer matrix (Figure 1c) at 0.5 wt% produces a transparent film with a finely structured absorption spectrum from 400 to 600 nm (Figure S1, Supporting Information) and a broad emission band from 500 to 700 nm centered at 580 nm (**Figure** **2**a). It should be noted that the mass concentration of rubrene converted to molar concentration is approximately 12 mm, which is sufficient for the occurrence of singlet fission and TTA.^[^ ^32^ ^]^ The emission lifetime of rubrene was estimated at 19.56 ns according to the time‐correlated single‐photon counting (TCSPC) measurements (Figure S2, Supporting Information), which are good for fast X‐ray irradiation detection. However, the weak X‐ray absorption cross‐section due to the lack of heavy atoms greatly limits its X‐ray sensitivity and imaging resolution. Although heavy‐atom modification of rubrene is one of the most effective methods to improve its X‐ray absorption cross‐section, the enhancement of non‐radiative transition due to the heavy atom effect and the huge amount of time and effort required for the synthesis make this method inefficient. In this case, we proposed an efficient energy transfer strategy by using a TADF chromophore with eight Br atoms (TADF‐Br) as the X‐ray sensitizer (energy donor) to improve the scintillation performance of rubrene (Figure 1b). Moreover, TADF‐Br exhibits near unity exciton utilization efficiency due to its minimized singlet‐triplet energy gap, leading to the efficient harnessing of both singlet and triplet excitons under X‐ray excitation. Therefore, the efficient energy transfer from TADF‐Br (D) to rubrene (A), the high X‐ray absorption cross‐section of D, and the near unity exciton utilization efficiency in both D and A significantly improved the X‐ray sensitivity and imaging resolution of rubrene.

TADF‐Br doped in PSF at 5 wt% has a broad absorption band from 300 to 500 nm and a strong emission band from 450 to 650 nm, which strongly overlaps with the absorption spectrum of rubrene (Figure 2a). The D–A*_n_* composite films (where *n* is the weight percentage of A, while D was embedded in PSF at a concentration of 5wt%) were engineered by adjusting the doping ratio of A to D (Figure 1c). As shown in Figure 2b, the emission intensity from D was quenched as the fraction of A gradually increased, which could be attributed to efficient energy transfer. At the same time, the photoluminescence (PL) lifetime monitored at 460 nm decreased from 2250 to 1190 ns (Table S1, Supporting Information), which further demonstrates the energy transfer from D to A (Figure 2c). It is worth noting that the drop of the donor emission can be due also to radiative energy transfer by absorption of photons emitted from the D by the A molecules (which is reasonable considering their absorption and emission properties). Therefore, the PL lifetime of the donor is not completely quenched.

The theoretical calculations were then performed to further investigate the energy transfer mechanism in these composite systems (Figure 2d; Figure S3, Supporting Information). As shown in Figure 2d, the highest occupied molecular orbital (HOMO) of D was consistently localized on the carbazole units, while the lowest unoccupied molecular orbital (LUMO) resided on the isophthalonitrile moiety. The well‐separated HOMO‐LUMO orbitals were consistent with its TADF characteristics. Moreover, the energy of both S_1_ and T_1_ states of D was higher than that of A, demonstrating the possibility of both singlet–singlet and triplet–triplet energy transfer from D to A. It is worth noting that the short D–A distance obtained from the DFT calculations (Figure S3, Supporting Information) further demonstrates the possibility of triplet–triplet energy transfer. In addition, the energy level of the S_1_ excited state of A was twice that of its triplet excited state, which further confirmed that the TTA could happen in this D–A system. The small energy gap of 0.16 eV between S_1_ and the ^1^(TT) pair states obtained from the calculation confirmed their fast interconversion, leading to the involvement of both processes in rubrene's light emission.

**Figure 3:**
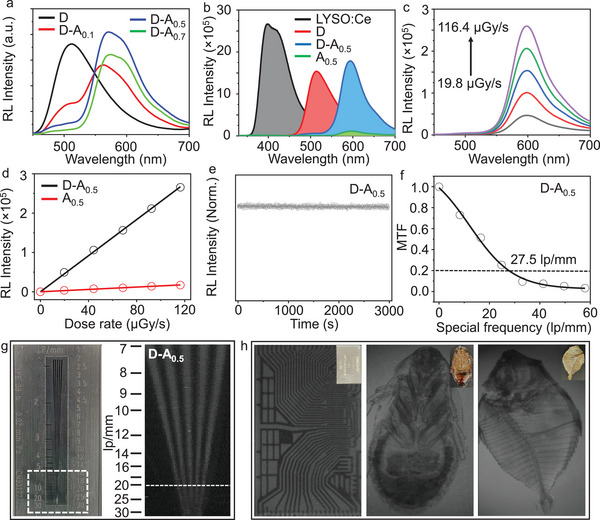
a) Radioluminescence (RL) spectra of the D–A*_n_* composite films (where *n* is the weight percentage of A, thickness: 0.25 mm). b) RL spectra of the standard scintillator LYSO:Ce, D, D–A_0.5_, and A_0.5_ films at the optimized thicknesses. c) The dose rate‐dependent RL spectra of the D–A_0.5_ composite film and d) the linear relationship between RL intensity and dose rate of D–A_0.5_ and A_0.5_ films at the optimized thickness (1.5 mm). e) RL intensity at emission maxima of D–A_0.5_ composite film under continuous X‐ray irradiation (dose rate, 1.26 mGy s^−1^). f) Modulation transfer functions (MTFs) of X‐ray images for D–A_0.5_ scintillation screen. Bright‐ and dark‐field photographs of g) a line‐pair card, h) an electronic chip (left), an insect (middle), and a fish (right) using D–A_0.5_ film as the X‐ray imaging screen.

The RL spectra of the D–A*_n_* composite films show the same trends as the corresponding PL spectra under UV excitation (**Figure** **3**a). It should be pointed out that the energy transfer should be the main factor leading to the enhancement of the RL from the acceptor because the luminescence of the donor is almost completely quenched no matter under the excitation of UV light or X‐ray. By analyzing the integrated area of the RL spectra of D–A_0.5_ and A_0.5_ films and comparing them with that of the standard scintillator LYSO:Ce (Figure 3b; Figure S4, Supporting Information), the light yield of the composite films can be roughly calculated. The D–A_0.5_ composite film has a high light yield of ≈20 000 photons per MeV, which is 11 times higher than that of the A film under the same experimental conditions (Table S2, Supporting Information). The RL intensity of the D–A_0.5_ and A_0.5_ films was linearly correlated with the dose rate of X‐rays (Figure 3c; Figure S5, Supporting Information), and the slope of the D–A_0.5_ composite film was ≈15 times higher than that of its A counterpart (Figure 3d). Moreover, the singlet fission‐based scintillator showed high photostability, and the RL intensity of the D–A_0.5_ composite film remained close to 100% over 3000 s of continuous ionizing irradiation at a dose rate of 1.26 mGy s^−1^ (Figure 3e), demonstrating the material's excellent photostability under X‐ray excitation.

More importantly, the fabricated D–A_0.5_ screen showed an ultra‐high X‐ray imaging resolution of 27.5 lp mm^−1^ (at a modulation transfer function [MTF] = 0.2), exceeding the resolution of most reported organic and commercial scintillators (Figure 3f; Table S3, Supporting Information). Moreover, this ultra‐high spatial resolution was further demonstrated by line‐pair card imaging, in which the line corresponding to >25 lp mm^−1^ (Figure 3g) could be recognized. Due to the excellent imaging resolution of the singlet fission‐based scintillation screen (D–A_0.5_), we performed a series of imaging tests to demonstrate their practical applications for use in high‐resolution X‐ray imaging. First, the steel spring inside a ballpoint pen under different loading states was well recognized, while the plastic shell showed a distinct color (Figure S6, Supporting Information). In addition, the application of this principle of X‐ray contrast imaging also enabled the inspection of the complicated inner structures of electronic devices. As shown in Figure 3h left, the inner structure of an electronic chip was visualized. More importantly, the transparent nature of the D–A_0.5_ composite screen was amenable to high‐resolution biological imaging. As shown in Figure 3h (middle and right), the skeletons of an insect and a fish were clearly observed under X‐ray exposure, making the scintillators excellent candidates for medical radiography and security screening.

## 3 Conclusion

We have developed a novel singlet fission‐based X‐ray imaging scintillator with high X‐ray sensitivity and spatial imaging resolution. The scintillator benefits from the efficient energy transfer from a TADF X‐ray sensitizer and the high exciton utilization efficiency in both X‐ray sensitizer (TADF‐Br) and singlet fission material (rubrene). The fabricated singlet fission‐based scintillator exhibits an ultra‐high X‐ray imaging resolution of 27.5 lp mm^−1^, exceeding that of most reported organic and commercial scintillators. This work verifies the feasibility of an energy transfer strategy for improving the X‐ray sensitivity of organic materials. Although the X‐ray absorption ability of TADF‐Br is not as good as that of inorganic materials, it provides a new way for the development of pure organic X‐ray imaging scintillators.

## Conflict of Interest

The authors declare no conflict of interest.

## Supporting information

Supporting Information

## Data Availability

The data that support the findings of this study are available from the corresponding author upon reasonable request.
