# Supplementary material for: Singlet Fission‐Based High‐Resolution X‐Ray Imaging Scintillation Screens
Source: Adv Sci (Weinh). 2023 Apr 21;10(19):2300406. doi: 10.1002/advs.202300406 (PMC10323610; doi:10.1002/advs.202300406)
Supplement: Supplementary file 1 — Supporting Information [file ADVS-10-2300406-s001.pdf]

ADVANCED SCIENCE
Open Access

# Supporting Information

for *Adv. Sci.*, DOI 10.1002/advs.202300406

Singlet Fission-Based High-Resolution X-Ray Imaging Scintillation Screens

*Jian-Xin Wang, Jun Yin, Luis Gutiérrez-Arzaluz, Simil Thomas, Wenyi Shao, Husam N. Alshareef, Mohamed Eddaoudi, Osman M. Bakr and Omar F. Mohammed**

# Singlet Fission-based High-Resolution X-ray Imaging Scintillation Screens

Jian-Xin Wang,[1] Jun Yin,[1,2,3] Luis Gutiérrez-Arzaluz,[1,2] Simil Thomas,[1,4] Wenyi Shao,[1] Husam N. Alshareef,[4] Mohamed Eddaoudi,[1] Osman M. Bakr,[2] and Omar F. Mohammed*[1]

[1]Advanced Membranes and Porous Materials Center, Division of Physical Science and Engineering, King Abdullah University of Science and Technology, Thuwal 23955-6900, Kingdom of Saudi Arabia

[2]KAUST Catalysis Center, Division of Physical Sciences and Engineering, King Abdullah University of Science and Technology, Thuwal 23955-6900, Kingdom of Saudi Arabia

[3]Department of Applied Physics, The Hong Kong Polytechnic University, 999077 Hong Kong, P. R. China

[4]Materials Science and Engineering, Physical Science and Engineering Division, King Abdullah University of Science and Technology (KAUST), Thuwal 23955-6900, Saudi Arabia

Correspondence: omar.abdelsaboor@kaust.edu.sa

**Materials and methods**

All chemicals were purchased from commercial suppliers and used without further purification. $^1$H NMR spectra were recorded on Bruker Avance III 400 MHz or 500 MHz instruments. UV-vis absorbance studies were carried out with PerkinElmer Lambda 950 UV/VIS Spectrometer. PerkinElmer LS45 Photoluminescence spectrometer having 450 W Xenon lamp was used for steady-state photoluminescence studies.

**Preparation of the scintillation screens.** 5 mg of TADF-Br chromophores were dissolved in 0.6 mL chloroform; n mg of rubrene was then added. After sonicating for 10 minutes in the dark, different amounts of polysulfone (PSF) were added. The mixture was sonicated for another 5 minutes and then shaken on a shaker for 5 hours to ensure that all materials were well mixed. The viscous solution was then carefully coated on the quartz plates to get the films for UV and X-ray correlated measurements. It is noteworthy that the films were covered with a beaker (covered with Aluminum foil) during the evaporation of the solvent to make sure the uniformity of the films.

**Time-Correlated Single-Photon Counting.** The time-resolved photoluminescence (PL) experiments for the samples were measured by the Time-Correlated Single-Photon Counting (TCSPC) technique. TCSPC measurements were performed in a Halcyone setup (Ultrafast Systems), and the corresponding excitation wavelengths were selected using a parametric optical amplifier (Newport, Spectra-Physics) that was pumped with an Astrella femtosecond pulsed laser (800 nm, 150 fs, 1 kHz, Coherent). Photoluminescence at different wavelengths was collected and recollimated by a pair of parabolic mirrors passed through a long-pass filter (422 nm, Newport) and finally focused on an optical fiber coupled to a monochromator and a PMT detector. The energy at each excitation wavelength was set constant with the help of a pair of variable neutral

density filters (Thorlabs) to ensure that less than 1 % of excitation events resulted in a detected photon. TCSPC histograms were fitted using the Lavenberg-Marquart algorithm implemented in Ultrafast System software. The overall time resolution for the system was better than 120 ps.[1, 2]

**Computational Methods.** The ground-state geometries of TADF-Br and Rubrene molecules were optimized using B3LYP functional together with 6-31G(d) basis set. The interfacial dimer structure for TADF-Br/Rubrene was built by placing two molecules together with an initial distance of ~3.0 Å and further optimizations were carried out. All the calculations were performed with Gaussian09 program (Rev D. 01).

The key intermediate in the singlet fission is the multiexciton ($^1$TT) state, a correlated triplet-triplet pair that facilitates the conversion of singlet exciton into two spin-triplet excitons. The singlet spin $^1$TT state is of double excitation nature, and this state cannot be described by typical TDDFT calculations and requires multireference methods to describe $^1$TT state. The excitation energy is calculated at the complete active space self-consistent field (CASSCF) level. Active space orbitals consist of two highest-occupied and two lowest-unoccupied molecular orbitals of the dimers, CASSCF(4,4). The calculations are carried out with a def2-TZVP basis set using the ORCA program.[3] The dimer geometry is taken from the experimental structure that displays a sizeable electronic coupling between rubrene dimers.

**Radioluminescence (RL) measurement.** Steady-state radioluminescence spectra were collected in a spectrometer (Fluoromax-4, Horiba) coupled with an X-ray tube (Tungsten target, Moxtex). The X-ray dose was controlled by tube current and tube voltage. By measuring the dose-dependent radioluminescence (RL) spectra, a linear relationship between the RL intensity against dose rate was obtained. A commercial scintillator LYSO:Ce (size: 1.5 × 1.5 cm, thickness: 500 µm, light

yield ~ 33000 photons/MeV) was used as a reference to estimate the light yield of the film samples. The films were fabricated to keep the same size as the LYSO:Ce wafer, and RL curves were obtained using the spectrometer under an identical configuration.

## Material synthesis

**General information.** All reactions were carried out with oven-dried glassware using standard Schlenk techniques under an inert atmosphere of dry argon or in an argon-filled glovebox. Analytical TLC was performed on Select Scientific 200 μm silica gel plates. Column chromatography was performed on Silicycle ultrapure silica gel with particle size 40-63 μm (230-400 mesh). All the solvents used were dried and distilled according to literature methods and degassed prior to use unless otherwise noted.

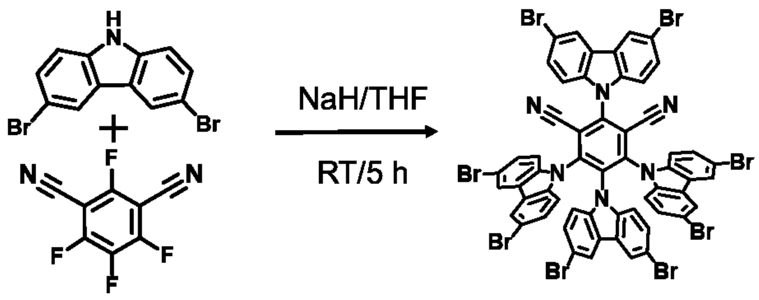


**Synthesis of TADF-Br.**[4] 3,6-dibromo-9*H*-Carbazole (4.5 mmol) was dissolved in 30 mL anhydrous THF in a two-neck flask. After bubbling with $N_2$ for 10 min, NaH (57-63% oil dispersion, 20.0 mmol) was added, and the mixture was allowed to stir at room temperature for 1h. The 2,4,5,6 tetrafluoro-1,3-dicyanobenzene (1 mmol) dissolved in 5 mL anhydrous THF was then slowly added, and the mixture was further stirred at room temperature for 5 h. The reaction was quenched with water under an ice bath, and the organic solvent was removed. 200 mL water was

then added and sonicated for 30 min. The crude product was obtained from filtration, which was further purified by recrystallization from acetone and fast column chromatography (DCM) to obtain the yellow powder. $^{1}H$ NMR (400 MHz, DMSO) δ 8.74 (d, $J$ = 1.7 Hz, 2 H), 8.30 (d, $J$ = 1.9 Hz, 4 H), 7.99 (ddd, $J$ = 13.3, 12.8, 5.3 Hz, 6 H), 7.65 (d, $J$ = 8.8 Hz, 4 H), 7.51 – 7.33 (m, 6 H), 7.05 (dd, $J$ = 8.8, 1.9 Hz, 2 H).

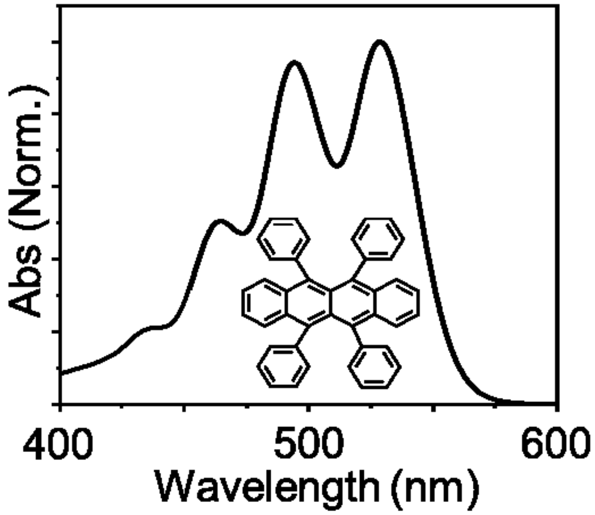


**Figure S1.** Absorption spectrum of rubrene doped in polysulfone (PSF) at 0.5 wt%.

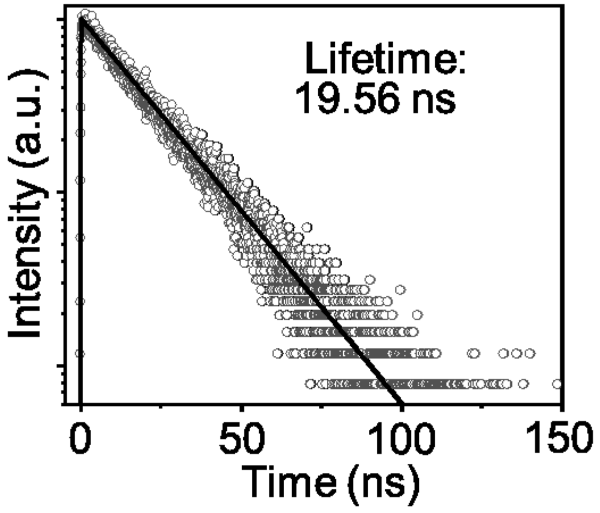


**Figure S2.** The PL decay profile of rubrene at the emission maxima doped in polysulfone (PSF) at 0.5 wt%.

**Table S1.** Collected lifetimes from the TCSPC measurements.

| Samples | Lifetimes (ns) |
|---|---|
| D @460 nm | 2250 |
| D-$A_{0.5}$ @460 nm | 1190 |

**Table S2.** Collected light yields of the optimized films.

| Samples | Light yields (photons/MeV) |
|---|---|
| $A_{0.5}$ | 1800 |
| D-$A_{0.5}$ | 19700 |

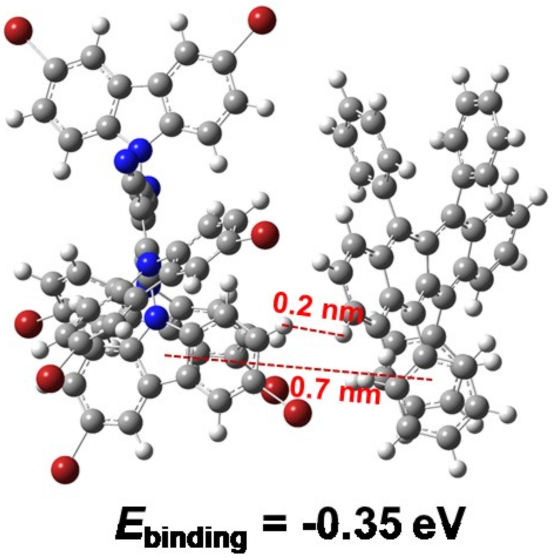


**Figure S3.** Optimized interfacial structure of the D/A interfaces.

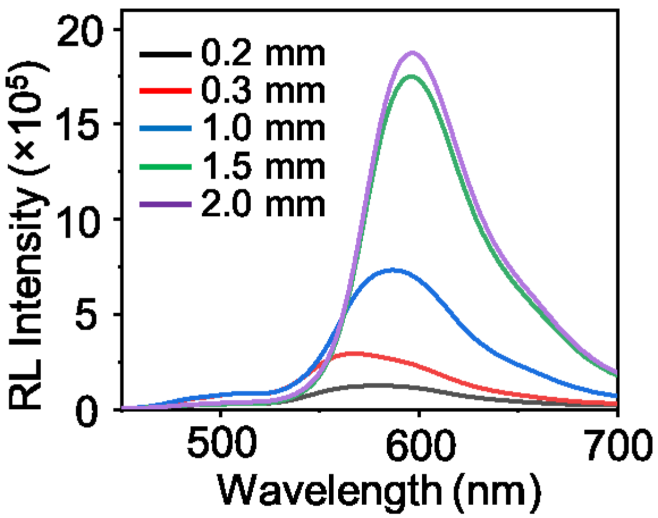


**Figure S4.** Radioluminescence spectra of the D-$A_{0.5}$ films at different thicknesses.

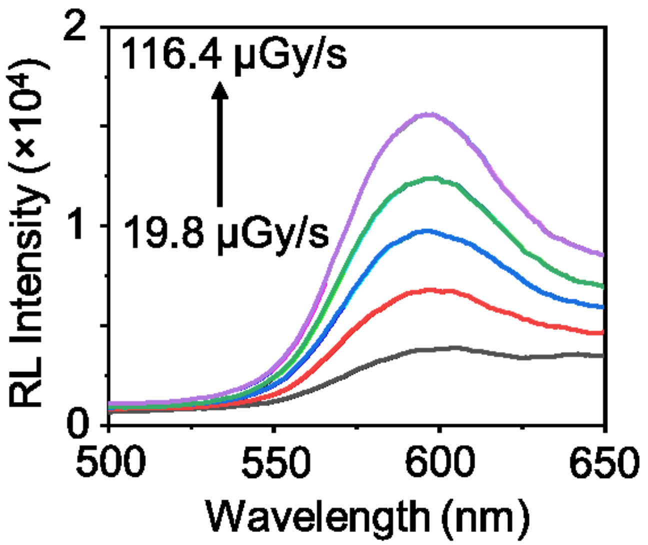


**Figure S5.** Dose rate-dependent radioluminescence spectra of $A_{0.5}$ film at the optimized thicknesses.

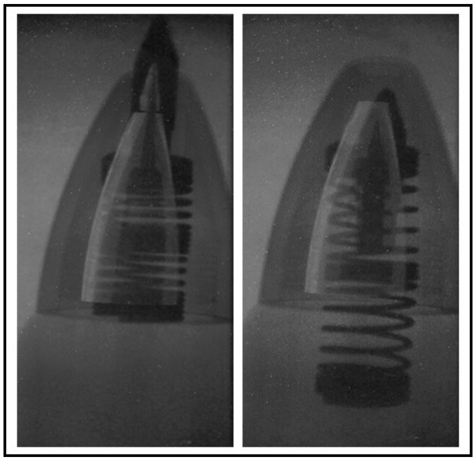

**Figure S6.** The X-ray imaging of a pen with a steel spring in different loading states using D-$A_{0.5}$ film as X-ray imaging screen.

**Table S3.** X-ray imaging resolutions of reported scintillators

| Materials | Imaging resolution | Ref. |
| --- | --- | --- |
| $Cs_3Cu_2I_5$/PDMS | 17 lp $mm^{-1}$ | [5] |
| DMAc-TRZ:SO scintillator screen | 16.6 lp $mm^{-1}$ | [6] |
| Cu-doped $Cs_2AgI_3$/PDMS | 16.2 lp $mm^{-1}$ | [7] |
| TPE-Br scintillation screen | 16.3 lp $mm^{-1}$ | [8] |
| In-doped $Cs_3Cu_2I_5$ single crystals | 18 lp $mm^{-1}$ | [9] |
| $BA_2PbBr_4$: Mn/PMMA | 10.7 lp $mm^{-1}$ | [10] |
| $TPP_2MnBr_4$ ceramics | 15.7 lp $mm^{-1}$ | [11] |
| TADF-Br – Ir-OMC composite scintillation screen | 19.8 lp $mm^{-1}$ | [12] |
| $CsPbBr_3$ NC/ceramic | 15 lp $mm^{-1}$ | [13] |
| $TEA_2MnI_4$ single crystal | 25 lp $mm^{-1}$ | [14] |
| NaLuF4:Tb/PDMS | 20 lp $mm^{-1}$ | [15] |

## References


[1] J.-X. Wang, Y. Wang, I. Nadinov, J. Yin, L. Gutierrez-Arzaluz, G. Healing, O. Alkhazragi, Y. Cheng, J. Jia, N. Alsadun, V. S. Kale, C. H. Kang, T. K. Ng, O. Shekhah, H. N. Alshareef, O. M. Bakr, M. Eddaoudi, B. S. Ooi, O. F. Mohammed, *J. Am. Chem. Soc.* **2022**, *144*, 6813-6820.

[2] J.-X. Wang, L. Gutiérrez- Arzaluz, J. Yin, P. Maity, Y. Zhou, C. Chen, Y. Han, O. M. Bakr, M. Eddaoudi, O. F. Mohammed, *Adv. Opt. Mater.* **2022**, *10*, 2200417.

[3] F. Neese, *Wiley Interdiscip. Rev. Comput. Mol. Sci.* **2012**, *2*, 73-78.

[4] H. S. Kim, J. Y. Lee, S. Shin, W. Jeong, S. H. Lee, S. Kim, J. Lee, M. C. Suh, S. Yoo, *Adv. Funct. Mater.* **2021**, *31*, 2104646.

[5]Y. Zhou, X. Wang, T. He, H. Yang, C. Yang, B. Shao, L. Gutiérrez-Arzaluz, O. M. Bakr, Y. Zhang, O. F. Mohammed, *ACS. Energy. Lett.* **2022**, *7*, 844.

[6]W. Ma, Y. Su, Q. Zhang, C. Deng, L. Pasquali, W. Zhu, Y. Tian, P. Ran, Z. Chen, G. Yang, G. Liang, T. Liu, H. Zhu, P. Huang, H. Zhong, K. Wang, S. Peng, J. Xia, H. Liu, X. Liu, Y. M. Yang, *Nat. Mater.* **2022**, *21*, 210-216.

[7]T. He, Y. Zhou, X. Wang, J. Yin, L. Gutiérrez-Arzaluz, J.-X. Wang, Y. Zhang, O. M. Bakr, O. F. Mohammed, *ACS. Energy. Lett.* **2022**, *7*, 2753.

[8]J.-X. Wang, Y. Wang, I. Nadinov, J. Yin, L. Gutiérrez-Arzaluz, O. Alkhazragi, T. He, T. K. Ng, M. Eddaoudi, H. N. Alshareef, O. M. Bakr, B. S. Ooi, O. F. Mohammed, *ACS. Mater. Lett.* **2022**, *4*, 1668-1675.

[9]Q. Wang, Q. Zhou, M. Nikl, J. Xiao, R. Kucerkova, A. Beitlerova, V. Babin, P. Prusa, V. Linhart, J. Wang, X. Wen, G. Niu, J. Tang, G. Ren, Y. Wu, *Adv. Opt. Mater.* **2022**, *10*, 2200304.

[10] W. Shao, X. Wang, Z. Zhang, J. Huang, Z. Han, S. Pi, Q. Xu, X. Zhang, X. Xia, H. Liang, *Adv. Opt. Mater.* **2022**, *10*, 2102282.

[11] K. Han, K. Sakhatskyi, J. Jin, Q. Zhang, M. V. Kovalenko, Z. Xia, *Adv. Mater.* **2022**, *34*, 2110420.

[12] J.-X. Wang, I. Dutta, J. Yin, T. He, L. Gutiérrez-Arzaluz, O. M. Bakr, M. Eddaoudi, K.-W. Huang, O. F. Mohammed, *Matter.* **2023**, *6*, 217-225.

[13] W. Ma, T. Jiang, Z. Yang, H. Zhang, Y. Su, Z. Chen, X. Chen, Y. Ma, W. Zhu, X. Yu, H. Zhu, J. Qiu, X. Liu, X. Xu, Y. M. Yang, *Adv. Sci.* **2021**, *8*, 2003728.

[14] Z.-Z. Zhang, J.-H. Wei, J.-B. Luo, X.-D. Wang, Z.-L. He, D.-B. Kuang, *ACS Appl. Mater. Interfaces.* **2022**, *14*, 47913.

[15] X. Ou, X. Qin, B. Huang, J. Zan, Q. Wu, Z. Hong, L. Xie, H. Bian, Z. Yi, X. Chen, *Nature.* **2021**, *590*, 410.
